# Visual data in health professions education: time to consider their use, ethics and aims

**DOI:** 10.1007/s40037-016-0279-z

**Published:** 2016-07-20

**Authors:** Elise Paradis, Patricia J. Leake

**Affiliations:** The Wilson Centre, University of Toronto and University Health Network, Toronto, Canada

In their Eye Opener on point of view (PoV) filming, Skinner and Gormley offer interesting insights into an innovative method of data collection [1]. According to the authors, this innovative method has two main affordances. First, PoV filming allows educators to see what trainees see, allowing the former to debrief with students asynchronously on a range of behaviours, especially around ‘people skills’. Videos can help students refine such skills through supervised examination, correction and praise. This feedback can lead to what the authors call ‘deep’, ‘immersive’ learning, even when the educator or supervisor is not co-present during activities. Second, PoV videos can be used as starting points during interviews, enabling interviewers to see with their own eyes what research participants experienced while wearing digital video glasses. Joint review of video footage by the interviewer and research participants enables the development of a greater shared understanding of participants’ experiences, can make the tacit visible, and ideally decolonizes the researchers’ gaze.

PoV filming is in many ways akin to other forms of data collection, such as the go-along method, the guided walk method, photovoice, and video-reflexive ethnography. To tease out its specificity, we need to consider its points of departure from these other methods.

The go-along [2–4] and guided walk [5] methods share the ethnographic sensibility of PoV filming and deeply value the experiences of research participants; they are part of the refle*x*ive turn in social research discussed by Skinner and Gormley in that they put participants’ experience at the centre of the research. Unlike PoV filming as described by the authors, however, these methods aim to underscore how behaviour is enabled, constrained and shaped by participants’ social contexts: access to resources, the built and lived environment, relationships with others, etc. Use of go-along methods is thus meant to help researchers appreciate the life of their research participants by sharing it with them, and enabling participants to verbalize how they experience the world. Empathy here is not developed by seeing through a participant’s eyes and discussing this perspective, but rather by experiencing the world with them. They are synchronous methods, whereas in PoV filming the observer is physically absent from the action, and thus observation is asynchronous.

Photovoice [6, 7] is another method that is gaining interest in health professions research, and it participates in both the refle*x*ive and the refle*ct*ive turns discussed by Skinner and Gormley. It is refle*x*ive in that it aims to give voice to the perspectives of participants on their own lives (and often disease) against an authoritative medical gaze. Participants choose what to emphasize. It is also refle*ct*ive: photovoice study participants take pictures of their life experiences, which they then share and discuss with researchers during individual interviews or focus groups. These pictures can transform perspectives of both researchers and participants.

Finally, video-reflexive ethnography [8–10] was developed to improve patient safety by transforming the culture of clinical workplaces through filming, debriefing and joint problem solving. It is a methodology that actively recognizes the power asymmetries between researcher and research participants, and thus values participants’ perspectives and goals in improving cultural practices. Video footage of real-life healthcare provision is used as the starting point for conversations about daily practices and their impact on safety and care quality. It has been used successfully to change hospital practices, but has not yet made inroads into undergraduate or graduate health professions education. PoV filming could easily be used as a tool to refine video-reflexive ethnography, and add another dimension, an alternative point of view from which to discuss clinical culture.

As researchers who are committed to making the voices of our participants heard and our research reflect their perspectives and experiences, we are excited that our field is using an increasingly broad range of qualitative research methods and methodologies. We believe that as most but not all individuals are immersed in the world visually, making use of visual modes of data collection and representation has the potential to illuminate new aspects of human experience. Yet many challenges remain for the use of visual data, be it photographs or video, particularly in published research.

First, as noted by the authors, comes the ethics of visual data collection. We owe it to our participants to protect them, their experiences and their identities, and we might not yet be aware of the range of ethical challenges that can arise out of such data collection efforts. Moreover, ethics boards are not yet equipped with good knowledge of the impact of image-based research on risk for participants. Along this dimension, go-along and guided-walk methods remain the safest, even as they are more time-consuming.

Second, if we take the primacy of the visual seriously, then using visual media merely as a means to elicit words – those of participants or of the researcher – continues to favour participants who are more skilled verbally. Visual methods have the potential to disrupt the pre-existing order, and to make the experiences of the less-verbal more visible and meaningful in research. How can we make sure that we realize this potential?

Third, in a publishing world that is focused on the written word, reporting non-verbal data is challenging. There are sometimes extra costs associated with publishing pictures, and most journals have limits on how many pictures they will publish per article. Videos are not yet a key modality in publishing and, if they are to be shared, will need to be hosted somewhere permanently.

Finally, we agree with the authors that it is important to decolonize research, but would like to argue that it is also critical that we decolonize health professions education. How can we use point of view and similar methods to transform medical education, and not just students or modes of content delivery? Where, we wonder, is the patient in PoV filming? How do we make sure that we listen and hear students as we try to teach them? How can we enable both patients and students to challenge us as educators and researchers who are in positions of authority?

In other words, we believe that the full potential of this approach might not be about finding the ‘true’ experience of research participants or tracing a ‘complete’ portrait of students’ experiences, but rather in disrupting what we take for granted as meaningful in, and for, health professions education.
